# Exploring Charge Transport Mechanisms and Dielectric Spectroscopy in Terp-Fc Layers Schottky Diodes under Temperature Variations

**DOI:** 10.1021/acsomega.5c00033

**Published:** 2025-05-08

**Authors:** Pınar Oruç, Ali Osman Tezcan, Serkan Eymur, Nihat Tuğluoğlu

**Affiliations:** a Department of Physics, Faculty of Sciences, Gazi University, Ankara 06340, Turkey; b Department of Electricity and Energy, Şebinkarahisar Vocational School, 187438 Giresun University, Giresun 28200, Turkey; c Department of Energy Systems Engineering, Faculty of Engineering, 187438 Giresun University, Giresun 28200, Turkey

## Abstract

This research proposed a novel Al/Terp-Fc/p-Si/Al diode configuration that has never been presented. In order to investigate how the temperature affects impedance spectroscopy and charge transport processes, we have constructed the Al/Terp-Fc/p-Si diode structure. We have also shown that the synthesized material has the potential to be applied to further research on organic semiconductors. Thermionic emission theory was used to evaluate the diode’s continual charge transport mechanism. In examining its functionality as a temperature sensor electronic device, we thoughtfully considered its ideality factor, barrier height, mobility, diffusion coefficient, diffusion length, and transit time. It is also observed that the reverse leakage current is dominated at all temperatures by Schottky emission at the measured voltage region. Low capacitance and high conductance values were noted in the manufactured Al/Terp-Fc/p-Si diode, especially at higher frequencies and at all temperatures. This phenomenon revealed the role of the temperature in the unique distribution, reorganization, and restructuring of surface states. The ac conductivity, dielectric constant, loss tangent, and complex dielectric/electric modulus of Al/Terp-Fc/p-Si diode were examined by the use of impedance/admittance characteristics at frequency range 300 Hz–1.5 MHz, operating between 300 and 400 K. Examining the dielectric characteristics’ frequency dependence revealed that all of these variables are frequency-dependent, particularly in the low-frequency range, which is caused by interface states and surface polarization. Furthermore, an analysis of the Cole–Cole diagrams of the Al/Terp-Fc/p-Si diode is conducted, and the corresponding equivalent circuit is deduced. The equivalent circuit of the fabricated diode consisted of a resistance (*R*_*g*_) and constant phase element (CPE_*g*_) connected with parallel. According to the results obtained, the designed device could be utilized as a capacitor in electrical circuits.

## Graphical abstract

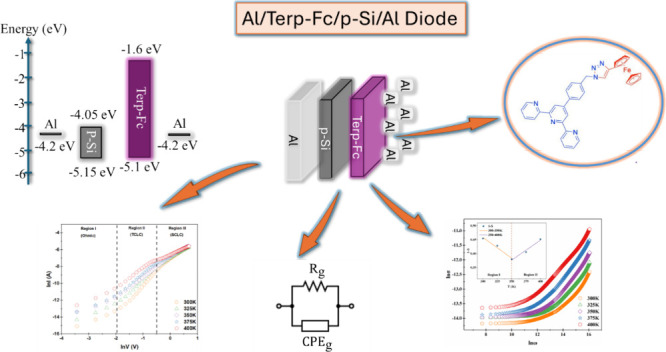

## 1 Introduction

In materials science, the exploration of electric and dielectric characteristics is paramount for understanding the fundamental behaviors of materials in various applications.^1–3^ Thin films have emerged as a captivating research area due to their unique properties, holding immense promise for advancements in electronics and optoelectronics.^4,5^ Their exceptional electrical activity makes them suitable for diverse applications such as capacitors, sensors, and photovoltaic devices, driving ongoing efforts to unravel their complex properties and harness their full potential in cutting-edge technologies.^6–8^

In electronics, the use of Ohmic and Schottky contacts is foundational and pivotal in the development of devices like field-effect transistors (FETs), solar cells, and photodetectors.^9,10^ Schottky diodes, essential building blocks in semiconductor technology, often feature crucial potential barriers at metal–semiconductor interfaces, influencing device performance and connectivity.^11–13^ Achieving consistent and thermally stable Schottky barrier heights (SBHs) remains a significant challenge in the fabrication of high-frequency and power Schottky diodes, underscoring the need for precise control and understanding of interface properties.^14–16^

Integration organic semiconductors into Schottky-type diodes represents a notable leap forward in electronic and optoelectronic applications.^17–20^ Unlike traditional inorganic materials, organic semiconductors offer distinct advantages, including tunable electronic properties, cost-effective fabrication processes, and compatibility with flexible substrates.^21,22^ Among the diverse chemical motifs studied, terpyridine and ferrocene stand out for their unique electrical characteristics and broad applicability across fields such as sensors, catalysis, and molecular electronics.^23–26^

This study marks a significant milestone with the synthesis and characterization of Terp-Fc, a novel material combining terpyridine and ferrocene components into a unified chemical structure. This innovative material holds promise for the development of multifunctional organic electronic devices. Herein, we explore Terp-Fc as an interfacial layer in Schottky diodes, meticulously examining its temperature- and frequency-dependent dielectric properties, modulus spectroscopy, and AC electrical conductivity through comprehensive experimental analysis.

Our investigation includes detailed impedance, dielectric, and modulus responses measured across an extensive frequency range (20 Hz to 1.5 MHz) and temperature range (300 to 375 K) for the Terp-Fc thin film device. We elucidate the material’s equivalent circuit model, dielectric constants, and relaxation dynamics, providing deeper insights into its electrical transport properties and potential applications in next-generation technologies.

Furthermore, our findings align with previous research, highlighting the significance of organic–inorganic semiconductor interfaces in enhancing the device performance and reliability. The incorporation of Terp-Fc into Schottky diodes enhances device functionality by mitigating leakage currents and improving charge injection and underscores its potential for achieving stable and reproducible high-barrier height interfaces.

In conclusion, this study sets the stage for further exploration of Terp-Fc-based organic electronic materials, positioning them as key enablers in advancing semiconductor technology. By integrating experimental insights with theoretical frameworks, our research aims to catalyze innovations in flexible electronics, sensor technologies, and beyond, harnessing the unique properties of organic semiconductors for future technological breakthroughs.

## 2 Experimental Details

### 2.1 Synthesis of Terp-Fc

The steps needed to be taken in synthesizing Terp-Fc are given in Scheme 1, which is new and has been introduced during this work. Compounds **3** (1.0 equiv) and **4** (1.0 equiv) were initially dissolved in a small amount of DMF. Then, the mixture was stirred continuously under an argon gas atmosphere until it became transparent. Next, sodium ascorbate (1.0 equiv) was added to the mixture, followed by a 5 min incubation under an argon atmosphere. Afterward, CuSO_4_·5H_2_O (1.0 equiv) was added to the mixture after another 5 min of exposure to an argon environment. The reaction mixture was stirred at 80 °C for 6 h after this. The precipitate was filtered, and the crude product was treated by using column chromatography. After that, a Terp-Fc-containing fraction was collected, and the solvent evaporated under reduced pressure. The white-colored product was synthesized with a yield of 80%. The ^1^H NMR (400 MHz, CHCl_3_): d 4.10 (s, 5H, Fc), 4.32 (s, 2H, Fc), 4.75 (s, 2H, Fc), 5.64 (s, 2H, CH_2_–N_3_), 7.48–7.34 (m, 5H, ArH), 7.99–7.85 (m, 4H, ArH), 8.69 (d, 2H, J = 7.9 Hz, ArH), 8.79–8.72 (m, 4H, ArH). ^13^C NMR (100 MHz, CHCl_3_): d 53.75, 68.68, 69.58, 75.28, 118.71, 118.92, 121.46, 124.02, 128.12, 128.51, 135.64, 137.11, 138.98, 147.44, 149.06, 149.41, 155.94.

**Scheme 1:**
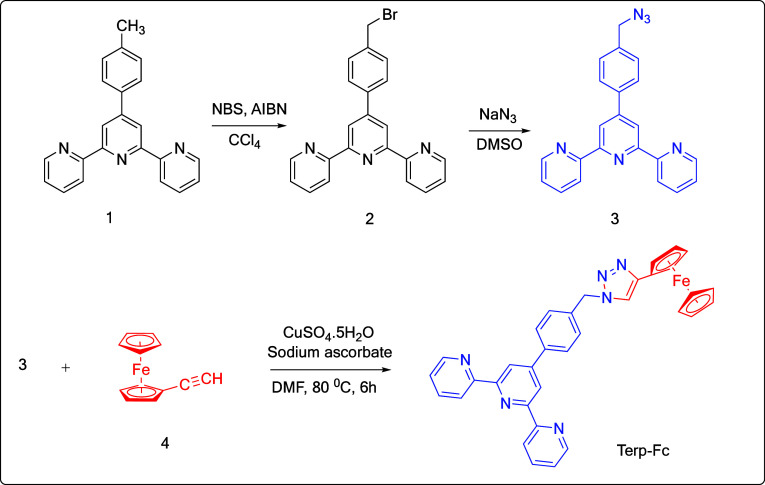
Synthetic Route to Terp-Fc Compound

### 2.2 Fabrication of Al/Terp-Fc/p-Si/Al Diode

A p-type Si (100) wafer was used to form the diode structure. The following sequence was applied to the p-type Si wafer. An RCA cleaning procedure was applied.^27^ A 200 nm thick Al ohmic contact was thermally evaporated on the unpolished side of the p-type Si wafer. A solution of the compound Terp-Fc in chloroform (CHCl_3_) was prepared and deposited on the polished p-Si wafer by the spin-coating process at 1500 rpm for 60 s. The film thickness is ∼55 nm. Finally, 200 nm thick Al rectifier contacts were thermally evaporated on the Terp-Fc using a 1 mm diameter circular metal mask. Consequently, as illustrated in Figure 1, an Al/Terp-Fc/p-Si/Al Schottky structure was fabricated. In order to accomplish this, the ITO substrate was subjected to an ultrasonic bath in a solution of chloroform, ethanol, acetone, and propanol for 5 min. Thereafter, Terp-Fc was cultivated on the ITO substrate by means of the spin-coating method, utilizing the same conditions as were employed for the fabrication of the diodes. The sample was then bisected into a uniform geometry, with silver contacts being obtained from each of the four corners. The electrical characteristics of the diode were analyzed using an HP 4192 A LF impedance analyzer system, measuring complex impedance frequency (*Z* – *f*), capacitance frequency (*C* – *f*), and conductance frequency (*G* – *f*) features. The study was performed at 300 Hz to 1.5 MHz frequencies and temperatures from 300 to 400 K with 25 K ranges. Using a Lake Shore model 331 temperature controller, a Keithley 2400 SourceMeter was used to measure current–voltage (*I*-*V*) throughout a temperature range of 300 to 400 K with 25 K ranges. The Hall measurement was taken at 0.5 T magnetic field and room temperature with the Van der Pauw technique using a Keithley 2400 SourceMeter and nanovolt meter.

**Figure 1:**
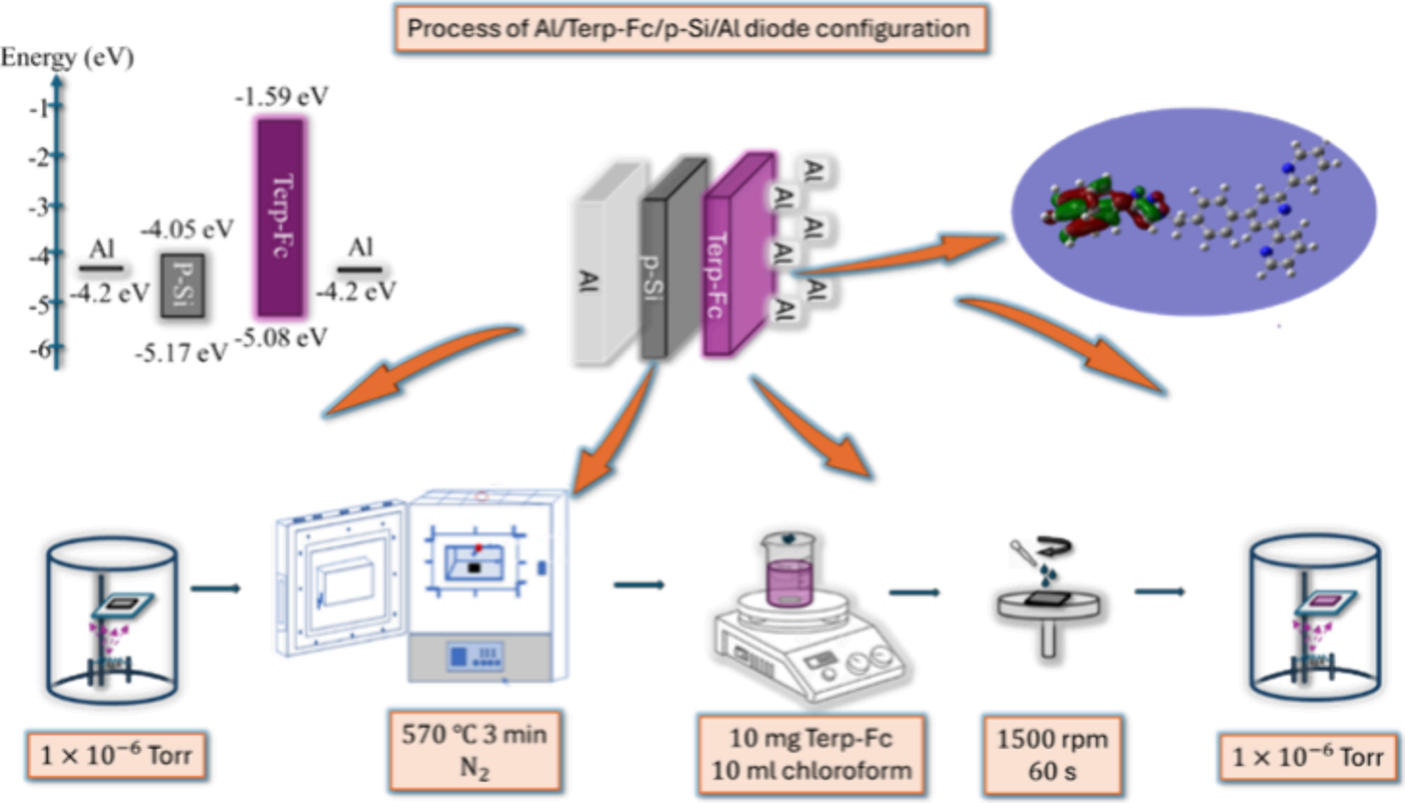
Illustration of the Al/Terp-Fc/p-Si diode’s production process.

Figure 2 shows a scanning electron microscope (SEM) image in cross-section. An average cross-sectional thickness of 50 to 53 nm is observed for the Terp-Fc organic compound on Si.

**Figure 2:**
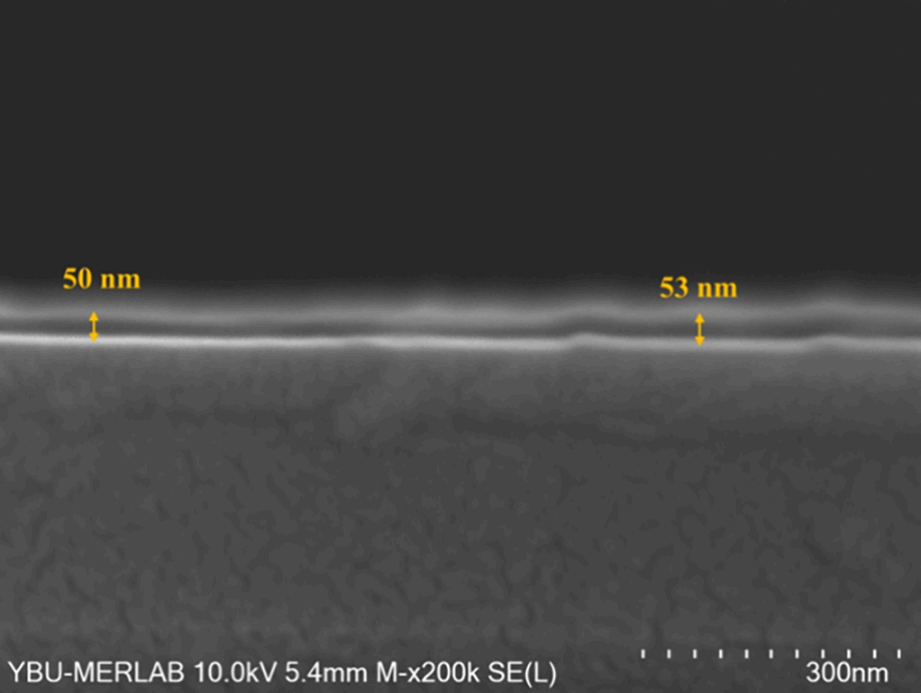
SEM image of cross-section showing the thickness (50 nm) of the Terp-Fc organic layer onto p-Si crystal.

## 3 Results and Discussion

To investigate the electronic properties of the organic Terp-Fc structure, a combination of experimental and theoretical approaches was employed, including Hall measurements and density functional theory (DFT) calculations. Hall measurements confirmed that the Terp-Fc material exhibits *n*-type semiconductor characteristics. The measurements revealed a negative Hall voltage, with a mobility of 166.65 cm^2^/V s and a sheet carrier density of 9.86 × 10^10^ cm^–2^ (Supporting Information, Section S2). These results indicate a moderate carrier transport efficiency and an appropriate free carrier concentration for electronic applications. Additionally, theoretical DFT calculations determined a HOMO–LUMO energy gap of 3.48 eV, suggesting that the material may exhibit semiconductor properties (Supporting Information, Figure S1 and Table S1).

### 3.1 Dielectric and Modulus Spectroscopies

In the dielectric spectroscopy section, we explained the dielectric properties of the fabricated Al/Terp-Fc/p-Si/Al diode. Figure 3a shows the graph of measurement capacitance versus frequency for different temperatures. Values of capacitance were decreased with increasing frequency for all temperatures. This result was observed because free-charge carriers cannot follow AC signals in high-frequency regions. When the dependence of capacitance on temperature is examined, it increases as the temperature increases. This state showed that the capacitance of Al/Terp-Fc/p-Si/Al depended on the temperature. This behavior is correlated with the generation of additional charge carriers with increasing temperature.^28,29^

**Figure 3:**
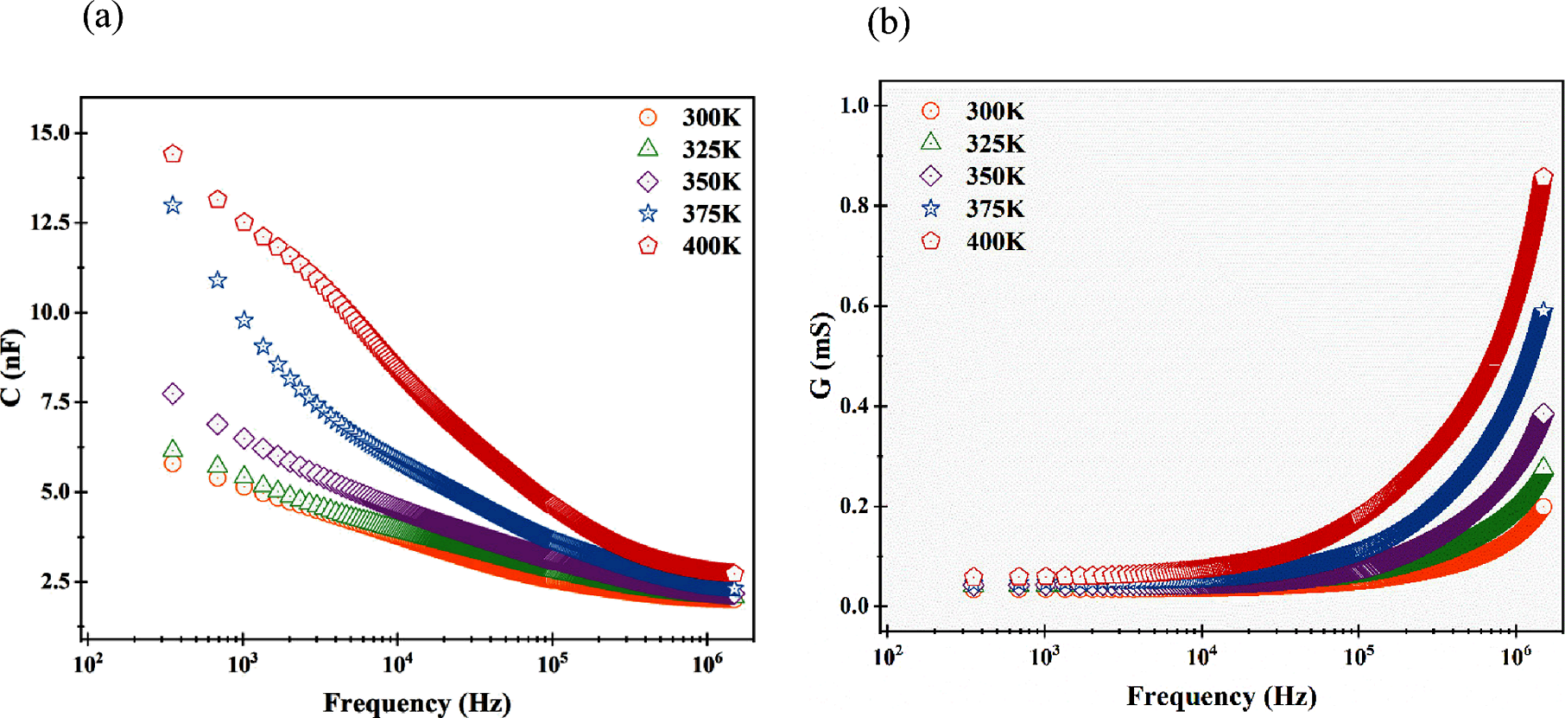
(a) Plot of capacitance versus frequency for the fabricated diode at different temperatures and (b) plot of conductance versus frequency for the fabricated diode at different temperatures.

The graph of conductance versus frequency for the fabricated diode is given in Figure 3b. Conductance values were almost steady at low-frequency regions, while they sharply increased with increasing frequency at high-frequency regions for all temperatures. These results can be because AC conductance is dominated at high-frequency regions, but DC conductance is dominated at low-frequency regions.^30^ When looking at the temperature dependence, conductance values increased with increasing temperature. This behavior is typically semiconductor behavior. Generally, the conductivity of semiconductor materials increases with increasing temperature because of the increasing generation of free-charge carriers with temperature.^31^ These results are in good agreement with other studies due to the results they showed.^32^

Changing the real part of the complex permittivity (ε′) with frequency is given in Figure 4a. ε′ is directly proportional to capacitance; it showed the same behaviors as the capacitance. ε′ decreased with increasing frequency and increased with increasing temperature. Values of ε′ changed with temperature between 1.34 for 300 K and 3.34 for 400 K at low-frequency regions, while they did not almost change at high-frequency regions. It may be evidence that free-charge carriers cannot follow AC signals at high-frequency regions. If the charge carriers have simple hopping processes, then net polarization can increase at low-frequency regions. ε′ increases with decreased frequency because of this case.^33^

**Figure 4:**
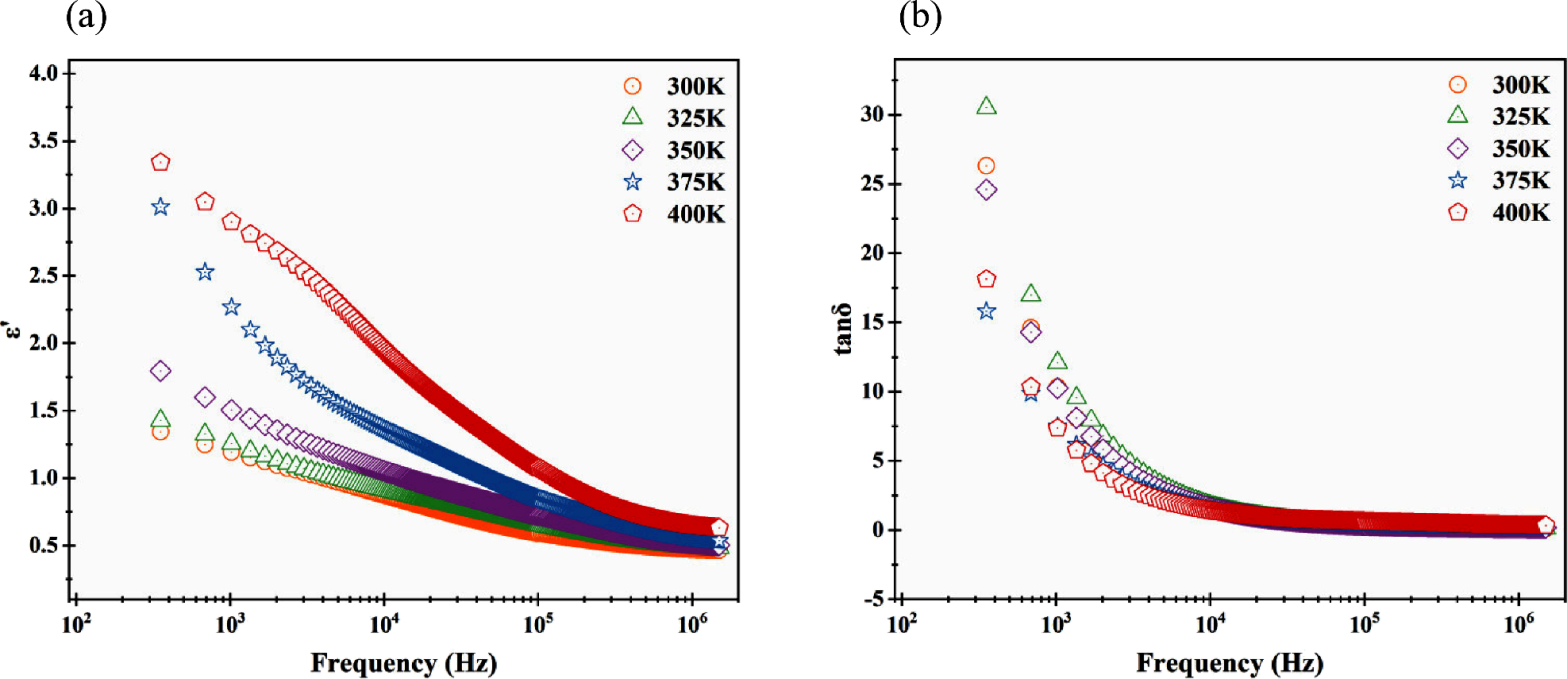
(a) Plot of the real part of complex permittivity versus frequency for the fabricated diode at different temperatures. (b) Plot of tanδ versus frequency for the fabricated diode at different temperatures.

The variation of tanδ with frequency is given in Figure 4b, and tanδ decreased with increasing frequency at high-frequency regions for all temperature values. Also, in the high-frequency region, tanδ saturated for all temperatures again. The tanδ reduced with the increased temperature at the low-frequency region for all temperatures. Also, any peaks were not observed. The similar behavior exists in previous literature.^5^

The relationship between the imaginary part of the dielectric constant and angular frequency is given in eq 1:^34^

$$\varepsilon^{\prime \prime }=A\omega^{m}$$ 1

*A* is a constant, *m* is the frequency power factor, and the value of it can be determined with the slope of the graph of ln ε″ versus lnω. The graph of ln ε″ versus lnω is given in Figure 5 for the fabricated diode at all temperature values. The values of *m* were negative at all temperatures, which were −0.65, −0.59, −0.51, −0.47, and −0.49 at 300, 325, 350, 375, and 400 K, respectively. The barrier height (*W*_*m*_) can be calculated with the help of eq 2:^34,35^

$$m=-\frac{4k_{b}T}{W_{m}}$$ 2

**Figure 5:**
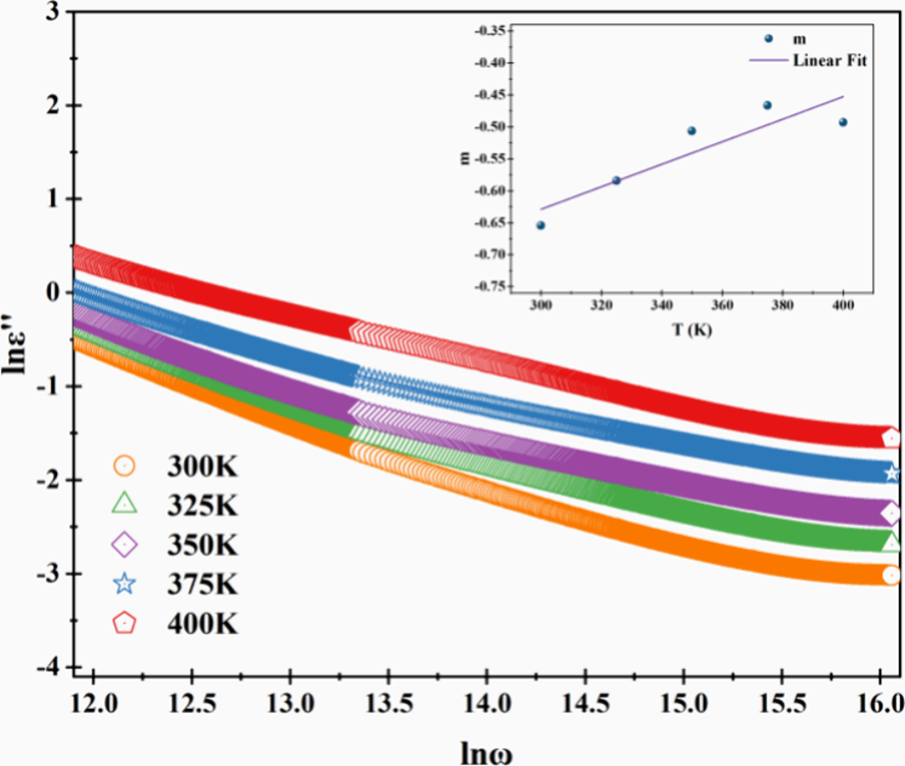
Plot of lnε″ versus lnω for the fabricated diode at different temperatures.

The barrier height is determined by the slope of the graph of *m* versus temperature, and it is given in Figure 5 inset. The barrier height is calculated as 0.19 eV with eq 2.

The graph in Figure 6a illustrates the frequency-dependent changes in the real component of the electrical modulus (*M*′). As frequency increased, the real part of the electrical modulus exhibited an upward trend, particularly accelerating in the midfrequency range while reaching a saturation point at both low- and high-frequency ranges. The consistent values observed in the modulus can be attributed to the high mobility of charge carriers and the minimal impact of the Maxwell–Wagner–Sillars polarization effect.^36^ Across all temperatures, the real part of the electrical modulus approached zero in the low-frequency region, reflecting the negligible impact of electrode polarization.^37^ Additionally, an increase in temperature led to a decline in the real part of the modulus, potentially explained by the short-range migration of charge carriers.^37^

**Figure 6:**
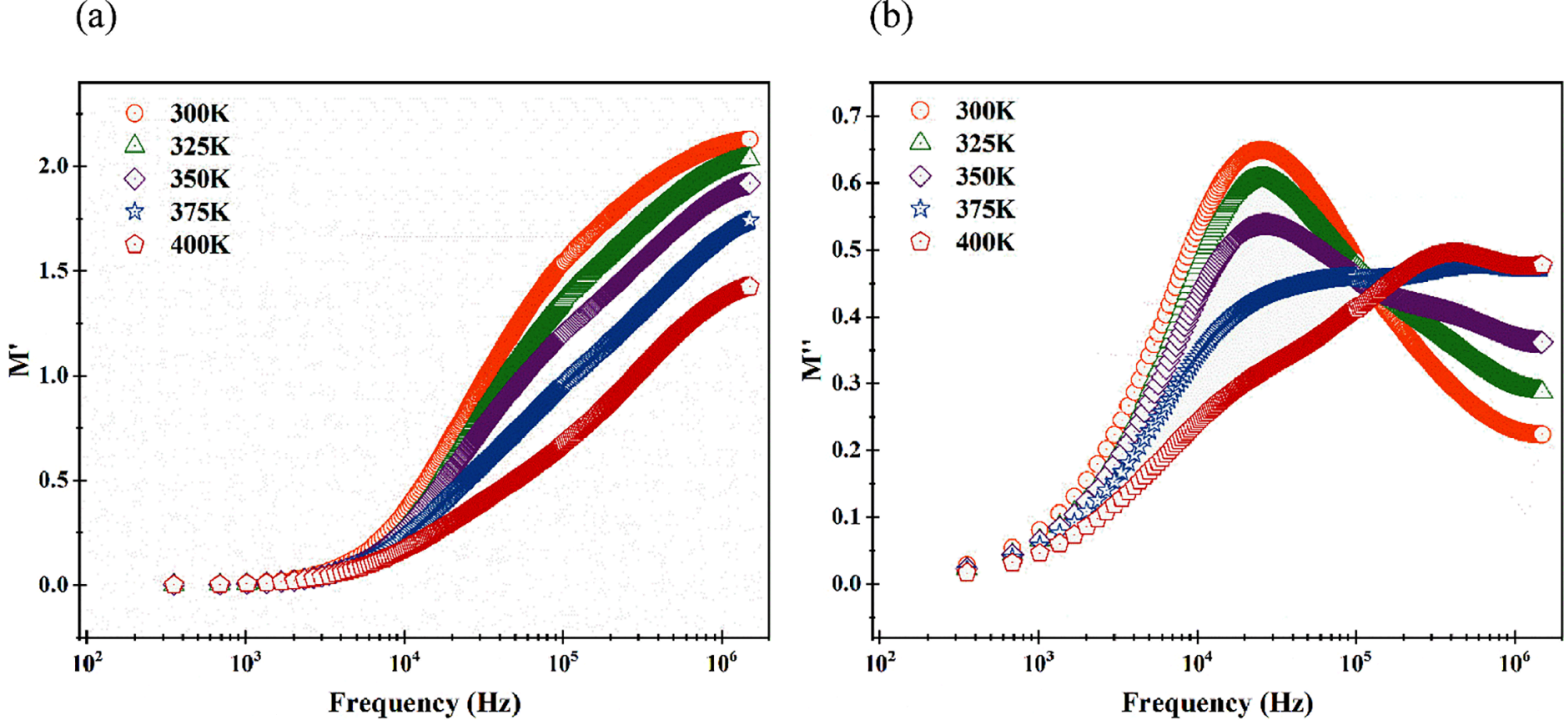
(a) Plot of the real part of modulus versus frequency for the fabricated diode at different temperatures. (b) Plot of the imaginary part of modulus versus frequency of the fabricated diode at different temperatures.

In Figure 6b, the imaginary part of the modulus (*M″*) is depicted against frequency for the fabricated diode at various temperatures. Notably, relaxation peaks were evident across all temperatures, exhibiting a shift toward higher-frequency regions. Simultaneously, the intensity of these peaks diminished and broadened as the temperature increased. Peaks in the low-frequency range signify long-term mobility of charge carriers, while those in the high-frequency range indicate short-term mobility. The observed shift of peaks to different frequency regions with temperature suggests a temperature-dependent nature of the relaxation processes. The corresponding relaxation times were 6.20 × 10^–6^, 6.20 × 10^–6^, 5.89 × 10^–6^, 7.17 × 10^–7^, and 3.76 × 10^–7^ s for temperatures of 300, 325, 350, 375, and 400 K, respectively.

### 3.2 AC Conductivity

Electrical conductivity (σ_AC_) is determined by eq 3:^38^

$$\sigma_{\mathrm{AC}}=A(T)\omega^{s(T)}$$ 3

The constant *A* is temperature-dependent, and the parameter *s* represents the frequency exponents. Parameter *s* assumes values between 0 and 1. The parameter *s* is associated with translation hopping. Figure 7 presents plots of lnσ_AC_ versus lnω at various temperatures. The s values were 0.55, 0.57, 0.62, 0.59, and 0.55 for 300, 325, 350, 375, and 400 K, respectively. First, they increased with rising temperatures; then, they decreased with rising temperatures. This trend aligns with the correlated barrier hopping (CBH) theory, which posits that *s* decreases as temperature increases, and nonoverlapping small polaron tunneling (NPST), which posits that *s* increases as temperature increases.^39^ The diode exhibits behavior consistent with the NPST theory between 300 and 350 K while demonstrating behavior in accordance with the CBH theory between 350 and 400 K. The relationship between the maximum-barrier height and *s* is defined in eq 4:^40^

$$s=1-\frac{6k_{b}T}{W_{m}}$$ 4

**Figure 7:**
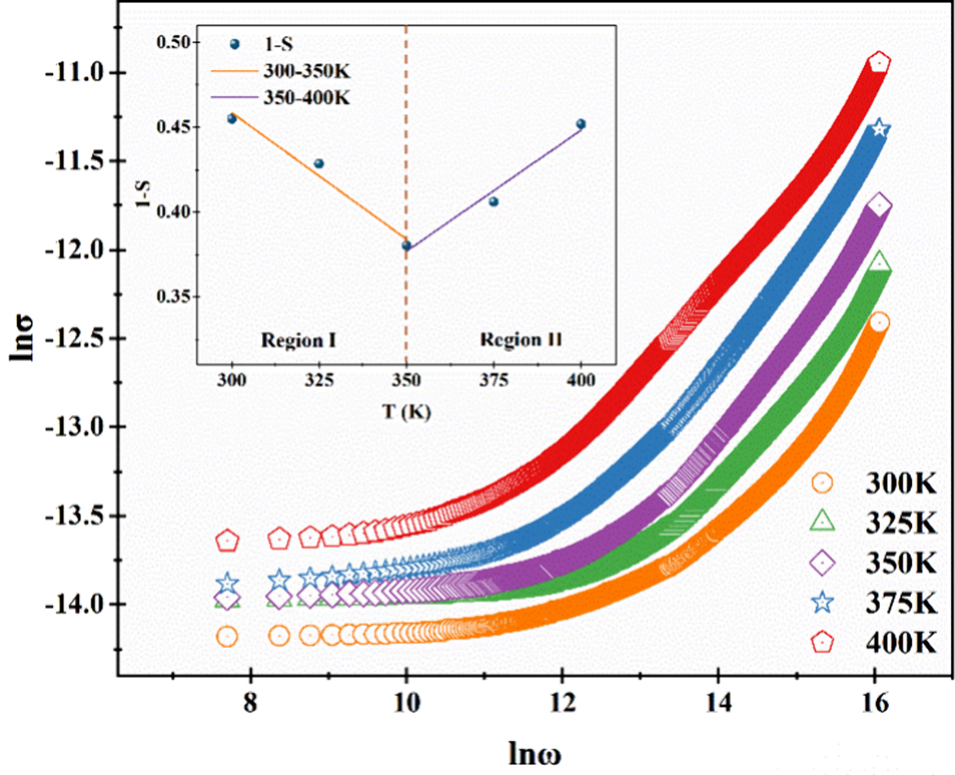
Plot of ln σ vs ln ω for the fabricated diode at different temperatures.

The graph of 1-s versus temperature is given in the inset of Figure 7 and values of *W_m_* were calculated as 0.345 eV for between 300 and 350 K and 0.369 eV for between 350 and 400 K.

### 3.3 Impedance Spectroscopy

The variation of the real part of the impedance with the frequency of the Al/Terp-Fc/p-Si/Al diode is shown in Figure 8a. According to this figure, *Z*′ decreased sharply with increasing frequency at the midfrequency region while in other frequency regions, it was almost steady with changing frequency for all temperature values. DC resistivity and the long-range movements of the charge carriers dominate in the low-frequency region. Also, in the high-frequency region, *Z*′ shows saturation behavior because of the resistive behavior of the grains of the materials. For these reasons, *Z*′ does not change with a changing frequency in the low- and high-frequency regions. On the other hand, the localized movement of the charge carriers and AC resistivity dominate in the middle-frequency region. Because of these reasons, *Z*′ depends on the frequency and decreases with increasing frequency.^41–43^ When the real part of the impedance depending on temperature was examined, it decreased with increasing temperature. According to this result, it may be said that the system has a negative temperature coefficient of resistance.^44^

**Figure 8:**
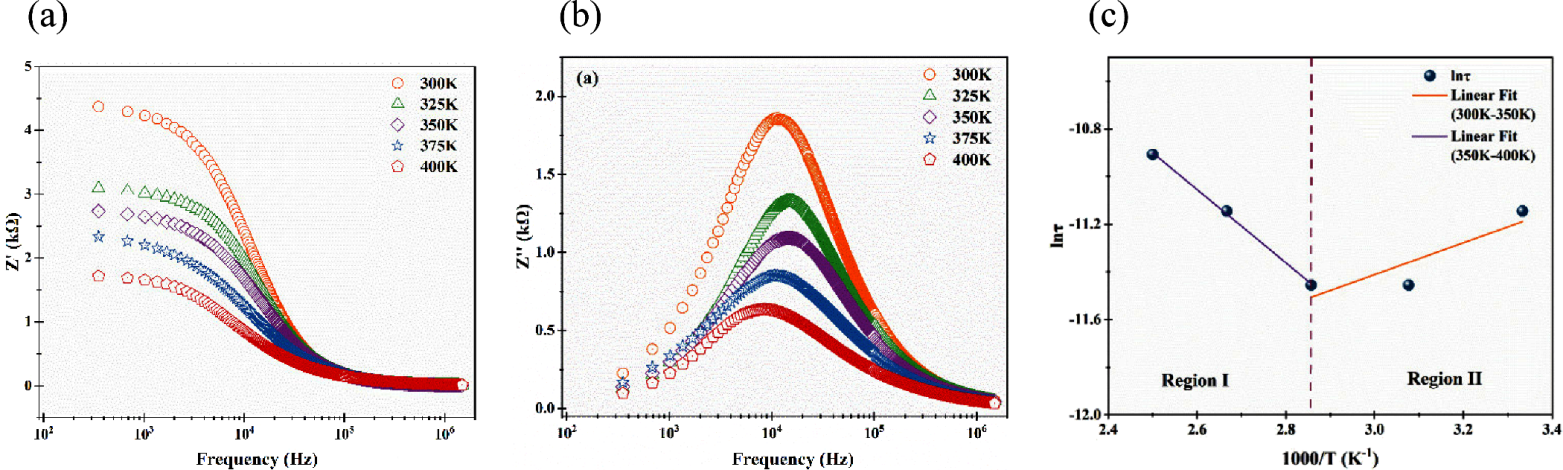
(a) Plots of the real part of impedance versus frequency for the fabricated diode at different temperatures. (b) Plots of the imaginary part of impedance versus frequency for the fabricated diode at different temperatures. (c) Plot of lnτ versus 1000/*T* for Terp-Fc/p-type Si/Al.

The graph of the imaginary part of impedance versus frequency of the fabricated diode is given in Figure 8b. The imaginary part of the impedance is defined as *Z*″ = *R* ^2^ *C*. Therefore, *Z*″ is the most resistive part in the impedance. One relaxation peak existed for all temperatures. According to Figure 8b, the peaks boarded with increasing temperature; this showed that the relaxation mechanism can depend on temperature and have multiple relaxation processes. The peaks between 300 and 350 K shifted toward the high-frequency region, while those between 350 and 400 K shifted toward the low-frequency region. This case might correspond to two relaxation processes, and the activation energies of each relaxation process were calculated to help the Arrhenius equality.

Using the peaks of relaxation processes, the activation energy was calculated. Relaxation times (τ) of peaks were determined by using the equivalation of τ = 1/*f*_max_ for each temperature. Figure 8c shows a graph of lnτ versus 1000/T for a fabricated diode from which activation energy (*E*_*a*_) was calculated using the Arrhenius equation:

$$\tau =\tau_{0}e^{E_{a}k_{b}T}$$ 5

According to Figure 8c, two different activation energies existed because of two different slopes. The activation energies of region I, which was between 350 and 400 K, and region II, which was between 300 and 350 K, were 0.13 and 0.57 eV, respectively. Also, the pre-exponential factors of region I and region II were 1.5 × 10^–6^ and 8.6 × 10^–4^ s, respectively. These two activation energies correspond to distinct carrier relaxation processes. The higher energy (0.57 eV) may be associated with deeper trap levels or interfacial states, while the lower energy (0.13 eV) indicates shallow trap-related relaxation dominating at higher temperatures. This dual nature implies that the Terp-Fc/p-Si interface supports efficient thermally activated charge transfer at elevated temperatures.

Nyquist plots of the Al/Terp-Fc/p-Si/Al diode are given in Figure 9 for all temperatures. One flatted semicircular was observed for all temperatures. The radii of these semicircular were decreased with increasing temperature, because the diode may have a behavior that is the negative temperature coefficient of resistivity. This is a typical behavior for semiconductor materials.

**Figure 9:**
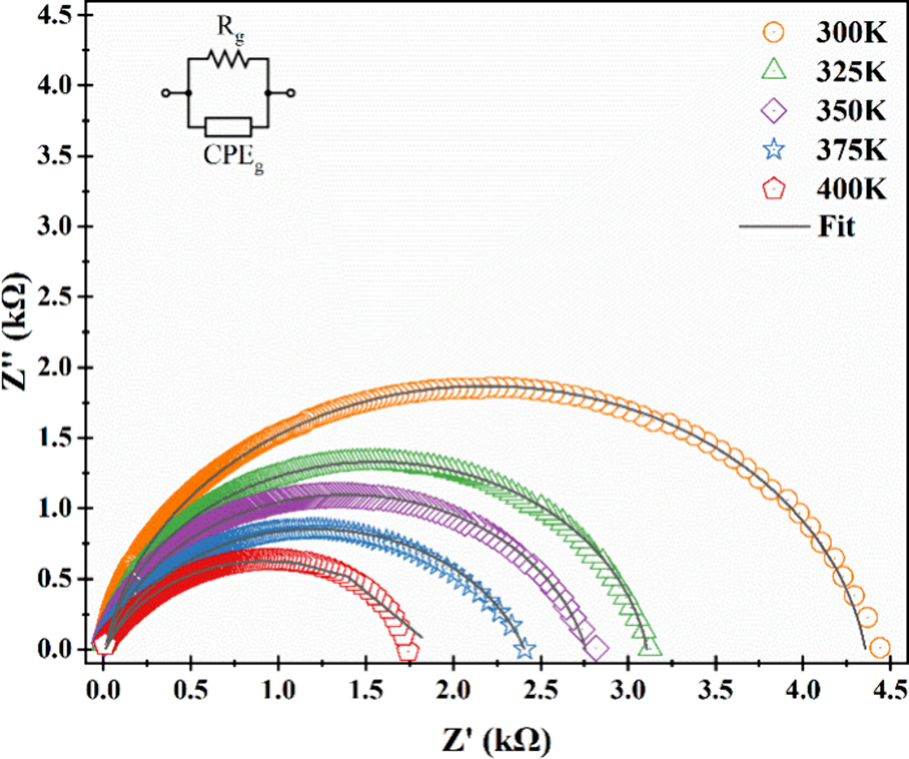
Plot of the real part of the impedance versus the imaginary part of the impedance for the fabricated diode at different temperatures.

Nyquist plots were fitted to a suitable equivalent circuit, and this scheme of the fabricated diode is given in Figure 9 inset. The circuit consisted of a resistance (*R*_*g*_) and constant phase element (CPE_*g*_) connected with parallel. The parallel circuit may describe the grain effect. They did not have any series resistance because of starting zero for all curves (Table 1). The equations of the equivalent circuit are given in eqs 6, 7, and 8:

$$Z=R_{g}\frac{A-iB}{A^{2}+B^{2}}$$ 6

$$Z^{\prime }=R_{g}\frac{A}{A^{2}+B^{2}}$$ 7

$$Z^{\prime \prime }=R_{g}\frac{B}{A^{2}+B^{2}}$$ 8

$$A=1+\omega^{n}R_{g}Q_{g}\cos\left(n\pi /2\right)B=\omega^{n}R_{g}Q_{g}\sin\left(n\pi /2\right)$$ 9

**Table 1:** Fitting Values of *R*_*g*_ *, Q*_*g*_ *,* and *n*

***T* (K)**	**300**	**325**	**350**	**375**	**400**
*R*_*g*_ × 10^3^ (Ω)	4.35	3.10	2.75	2.40	1.85
*Q*_*g*_ × 10^–9^ (F)	6.00	8.40	2.00	4.50	7.00
*n*	0.905	0.905	0.860	0.790	0.765

The values of all circuit elements fitted according to the analytical equation for each temperature are given in Table 2. The grain resistance (*R*_*g*_) decreased, and grain *Q*_*g*_ and *n* decreased with increased temperatures. According to these results, we can say that the CPE showed nearly perfect capacitance behaviors at lower temperatures, such as 300 and 325 K because of the values of approximately 1 *n*. Also, in light of this information, we can say that the effect of the grain decreased with increasing temperature ().

**Table 2:** Summary of the Experimental Parameters of the Terp-Fc/p-Si Diode at Different Temperatures

		I–V			*dV*/*d*(*ln I*) – *I H*(*I*) – *I*			
temperature (K)	RR	*I*_0_ (nA)	*n*	Φ_*b*_ (eV)	*R*_*s*_ (Ω)	*n*	Φ_*b*_ (eV)	*R*_*s*_ (Ω)
300	600	296.5	2.87	0.704	306.9	6.87	0.589	299.0
325	312	700.9	2.64	0.743	302.0	7.38	0.577	306.7
350	216	1411	2.35	0.783	302.9	4.98	0.614	295.6
375	135	3021	2.24	0.819	324.1	6.96	0.571	316.1
400	70	3722	2.02	0.871	326.9	8.69	0.548	317.7

### 3.4 Current Voltage Characteristic for Terp-Fc/p-Si Schottky Diode

The temperature of 300, 325, 350, 375, and 400 K for the Terp-Fc/p-Si diode was used to determine the current–voltage (*I*-*V*) characteristics to study the changes in the Schottky barrier diode parameters. Figure 10 displays the reverse and forward bias characteristics of the Terp-Fc/p-Si diode at temperatures of 300, 325, 350, 375, and 400 K. It is easily apparent that the Schottky diode is temperature-dependent and has good rectifying properties at the measured temperatures. All the curves in Figure 10 show a rectification behavior, and the rectification rate (RR) in the 300 K is high at ± 4 V, with a value of 600. Table 3 provides the rectification rates at ± 4 V for all temperatures. As the TE model estimates, an increase in the current in Figure 10 is seen as the temperature rises. The relationship between the reduction in the voltage falling on the device (*V*_*d*_ = *V* – *IR*_*s*_) and the current (*I*) in the TE model is commonly written as follows:^45,46^

$$I=I_{0}\left[\exp\left(\frac{q\left(V-IR_{s}\right)}{nkT}\right)-1\right];I_{0}=AA^{*}T^{2}\exp\left(-\frac{q\Phi_{b}}{kT}\right)$$ 10

**Figure 10:**
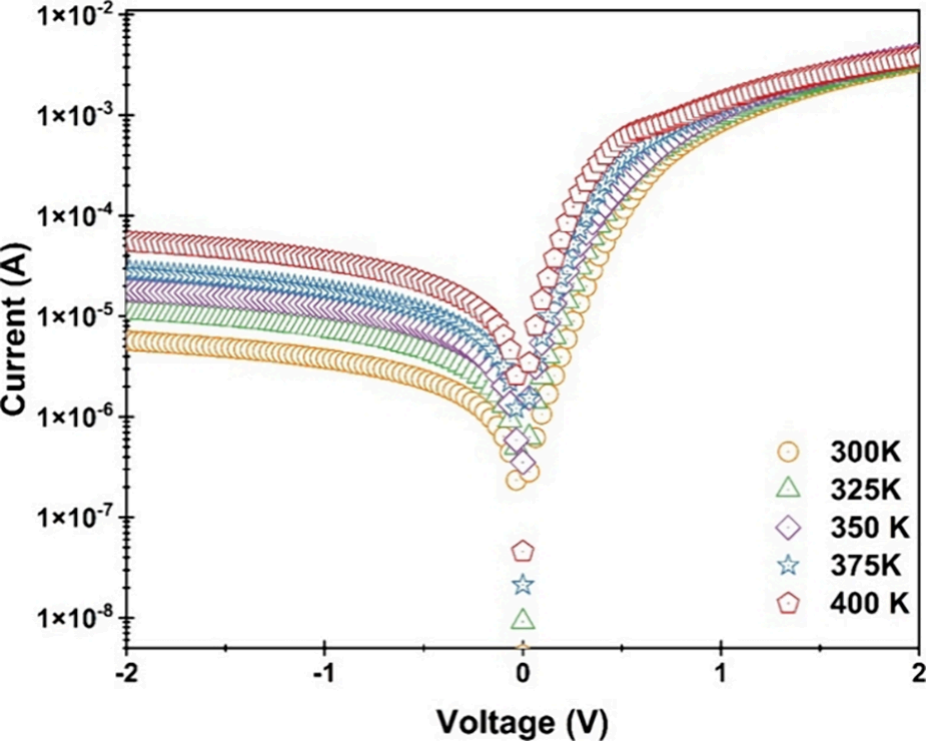
ln*I*-*V* graphs of the Al/Terp-Fc/p-Si diode in the temperature range from 300 to 400 K.

**Table 3:** Experimental Values of Charge Transport Parameters of Al/Terp-Fc/p-Si Diode at Different Temperatures

temperature (K)	β (eV cm^1/2^ V^–1/2^)	μ_eff_ (m^2^ V^–1^ s^–1^)	τ (s)	*D* (unitless)	*L_D_* (m)	σ (S m^1–^)	*N* (eV cm^–3^)
300	6.12 × 10^–5^	7.72 × 10^–14^	1.87 × 10^–5^	2.00 × 10^–15^	2.74 × 10^–10^	8.21 × 10^–13^	6.65 × 10^19^
325	6.16 × 10^–5^	7.67 × 10^–14^	1.99 × 10^–6^	2.33 × 10^–15^	9.62 × 10^–11^	16.4 × 10^–13^	13.4 × 10^19^
350	5.13 × 10^–5^	8.16 × 10^–14^	2.01 × 10^–5^	2.46 × 10^–15^	3.14 × 10^–10^	24.6 × 10^–13^	18.9 × 10^19^
375	7.76 × 10^–5^	8.05 × 10^–13^	1.98 × 10^–5^	2.60 × 10^–14^	1.02 × 10^–9^	49.3 × 10^–13^	38.3 × 10^19^
400	7.93 × 10^–5^	7.72 × 10^–13^	1.91 × 10^–5^	2.66 × 10^–14^	1.01 × 10^–9^	82.1 × 10^–13^	66.5 × 10^19^

*R*_*s*_ is the series resistance, Φ_*b*_ is Schottky barrier height, *I*_0_ is saturation current, and *n* is the dimensionless ideality factor. The diode’s Φ_*b*_ and *n* values determined with TE theory are listed in Table 2. At increasing temperatures, it can be seen in Table 2 that the Φ_*b*_ values grow and the *n* values (more than 1) drop. As is well known, the *n* values ought to be equal to or extremely near to 1 in accordance with the classical TE theory. In the same way that the forbidden energy gap reduces as the temperature rises, the Φ_*b*_ values are also predicted to do so. This situation shows that it deviates from the TE theory. According to TE theory, although it is known that the barrier height is constant in metal/semiconductor (MS) and metal/interfacial layer/semiconductor (MIS) structures, many researchers have reported studies on the barrier not being homogeneous.^47–51^ The existence of interface states, barrier inhomogeneity, and the influence of the interfacial layer were found to be the causes of the observation that *n* values decreased with temperature while Φ_*b*_ values increased.

The series resistance (*R*_*s*_) in the MS and MIS structure is one of the crucial electrical characteristics. The series resistance effect is more noticeable at high direct voltage values, and the diode’s *I*-*V* characteristic exhibits a more noticeable deviation from linearity. The Cheung approach is a further technique for estimating *R_s_* in the MS and MIS systems.

Figure 11b shows the graphs of *dV*/*d ln*(*I*) versus *I* and *H*(*I*) versus *I* for all of the measured temperatures, respectively. Figure 11a,b is used to derive the *n* and *R*_*s*_ and Φ_*b*_ values, which are shown in Table 2 for all measurement temperatures.

**Figure 11:**
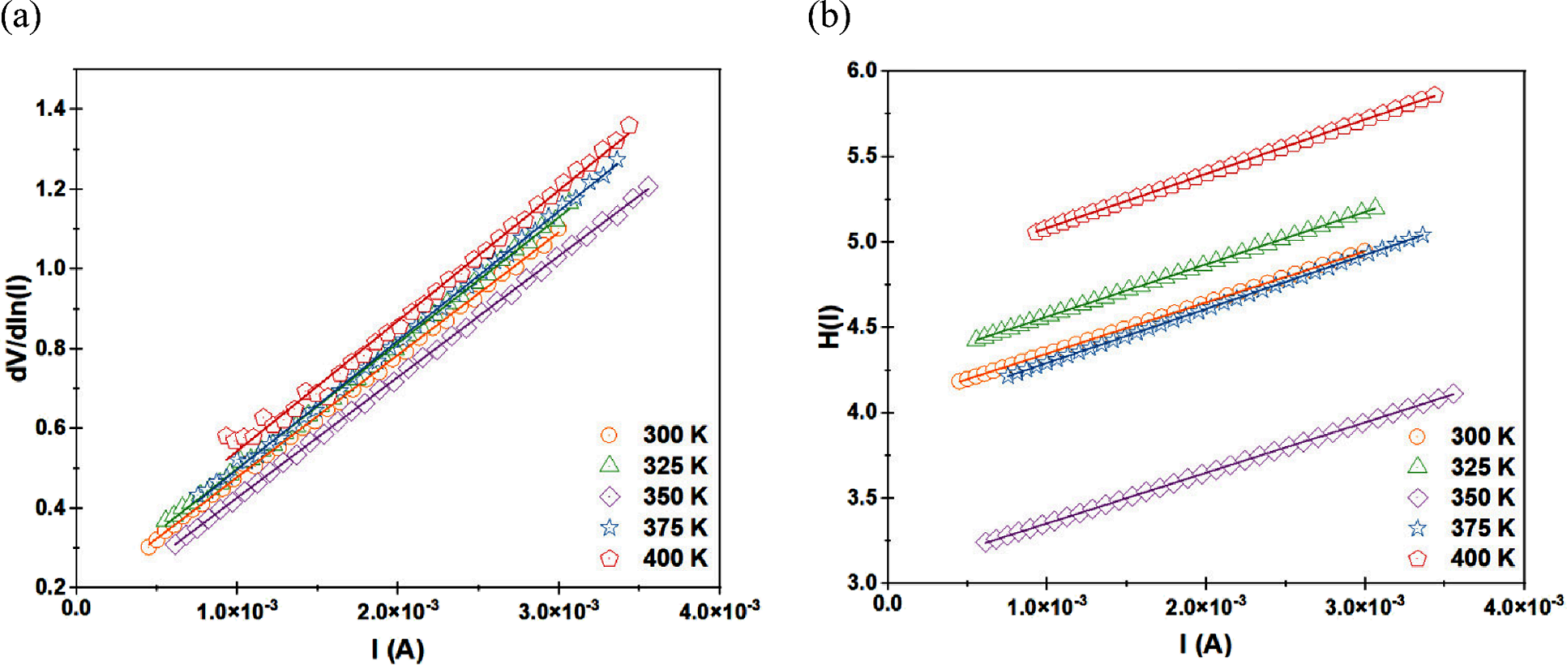
For the temperature range from 300 to 400 K, (a) *dV*/*d ln*(*I*) versus *I* graphs and (b) *H*(*I*) versus *I* graphs.

To better comprehend the current transport features of the Terp-Fc/p-Si diode, its *I*-*V* data was shown on a log–log scale. The log *I*–log *V* curves of the Terp-Fc/p-Si diode determined at different temperatures are shown in Figure 12. Ohmic regions have a slope of *m* = 1, space charge limited current (SCLC) regions have a slope of *m* = 2, and trapped charge restricted current (TCLC) regions have a slope of *m* > 2. Figure 12 indicates that the *logI–logV* plots for the Terp-Fc/p-Si diode exhibit three distinct linear regions, suggesting the presence of different conduction processes, where the diode current follows an *I*–*V* ^*m*^ relationship and the *m* values for all measured temperatures are determined from the slopes of these regions.

**Figure 12:**
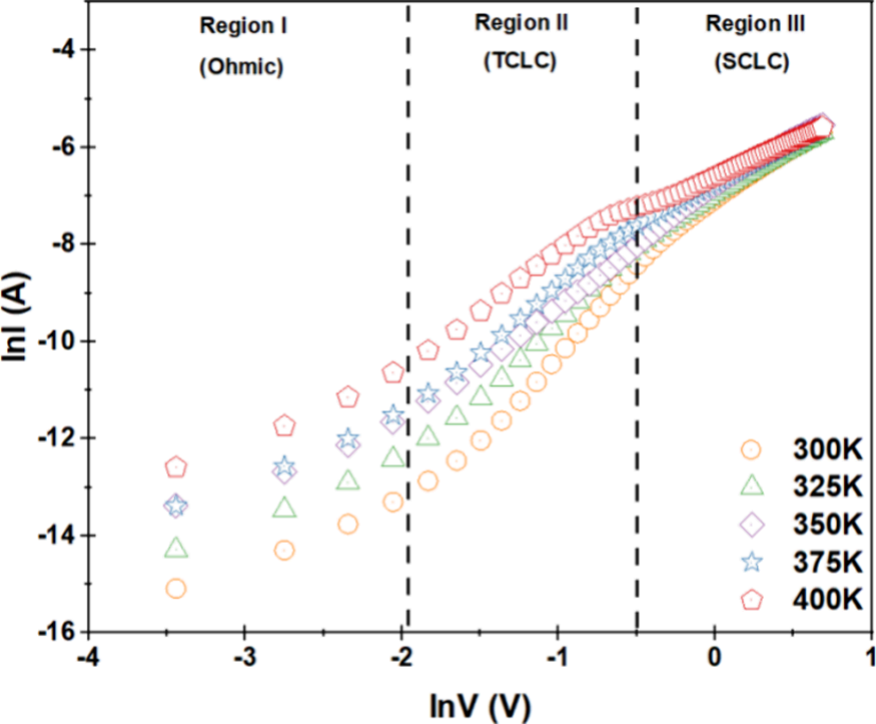
*ln*(*I*) versus *ln V* graphs of Al/Terp-Fc/p-Si diode in the temperature range from 300 to 400 K.

The ranges that were used to establish the first, second, and third regions were 0.032–0.128, 0.16–0.64, and 0.672–2.0 V, respectively. The Terp-Fc/p-Si diode’s first, second, and third regions represent ohmic, TCLC, and SCLC current transport behavior, respectively. For example, the *m* values for 1, 2, and 3 regions were determined to be 1.27, 3.48, and 2.26 at 300 K and 1.27, 3.48, and 2.26 at a temperature of 400 K, respectively.

A reverse current analysis provides an array of information regarding the Terp-Fc/p-Si interface. We examined the reverse leakage current of the Terp-Fc/p-Si diode by considering the Schottky emission (SE) and Poole-Frenkel emission (PFE). The SE is connected to the reverse current (*I*_*R*_), which is expressed by^52^

$$I_{R}=AA^{*}T^{2}\exp\left(\frac{-\Phi_{b}}{kT}\right)\exp\left(\frac{\beta_{SE}V^{1/2}}{kTd^{1/2}}\right)$$ 11

where β_PFE_ and β_SE_ are the Poole-Frenkel’s field-lowering coefficient according to the PFE mechanism, and the Schottky’s field-lowering coefficient according to the SE mechanism, respectively. The following relation is used to determine the theoretical values of β_PFE_ and β_SE_:^53^

$$\beta_{PFE}=2\beta_{SE}={\left(\frac{q^{3}}{\pi \varepsilon_{0}\varepsilon_{r}}\right)}^{1/2}$$ 12

The β_PFE_ value is consistently double the β_SE_ value. For the Al/Terp-Fc/p-Si diode, the theoretically computed values of β_PFE_ and β_SE_ are approximately 4.34 × 10^–4^ and 2.17 × 10^–4^ eV cm^1/2^ V^–1/2^, respectively.

As illustrated in Figure 13a, the graph of current density versus voltage in the reverse feed region demonstrates a positive correlation between the temperature and leakage current. Utilizing the reverse bias region (Figure 13b), the ln­(*J*_*r*_ */T* ^*2*^) versus *E*_*r*_^0.5^ plot yielded linear graphs with positive slopes across all temperatures. The linearity and positive slope of the graph indicate the dominance of the Schottky effect in all temperature regions of the diode. Also, the observed upward shift in the curves with respect to temperature indicates that the increase in current with temperature is in accordance with the expected relationship.^54–56^ The β values were calculated by taking the slope for all temperature values and are presented in Table 3. A comparison of the results obtained from Table 3 with the theoretically calculated results indicates the existence of the Schottky effect.

**Figure 13:**
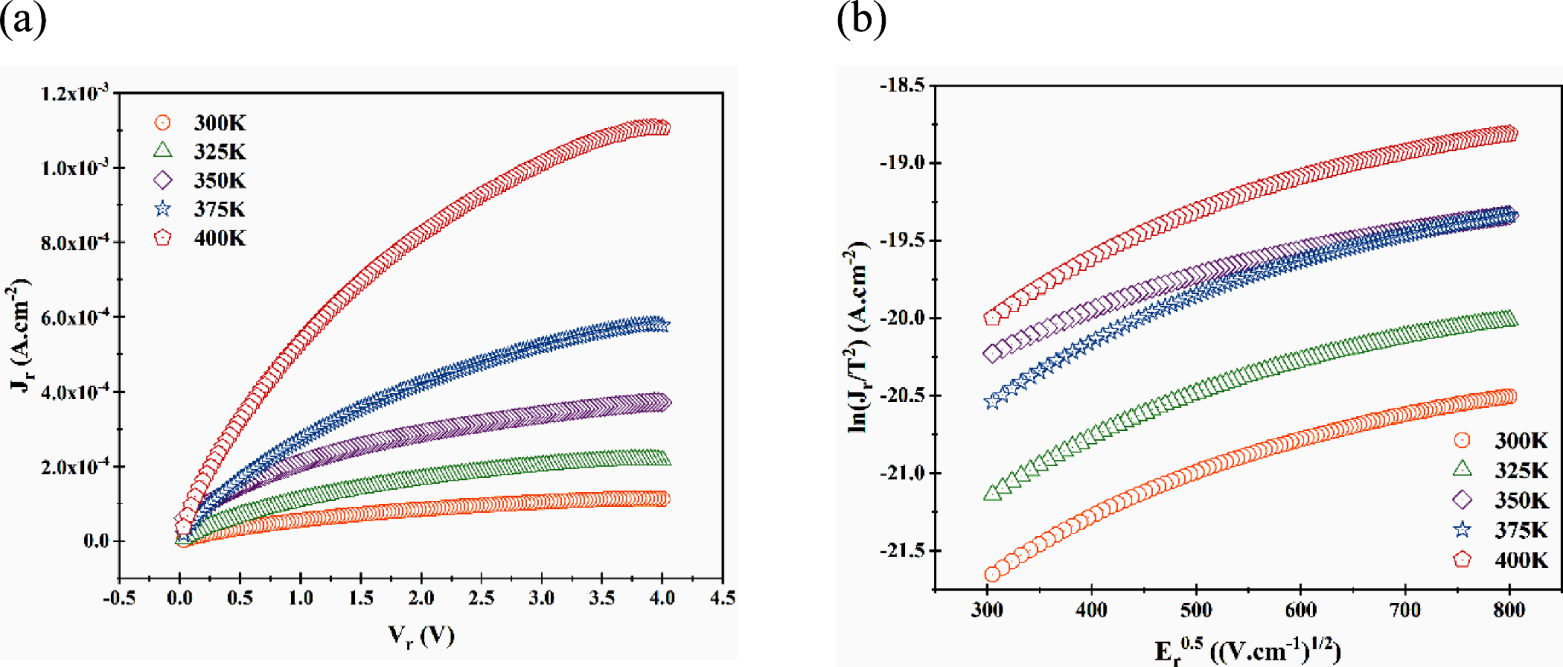
(a) *J*_*r*_ versus *V*_*r*_ and (b) ln­(*J*_*r*_ */T* ^*2*^) versus *E*_*r*_^0.5^ graphs of Al/Terp-Fc/p-Si diode in the temperature range from 300 to 400 K.

One of the best areas to determine the charge conduction characteristics is the SCLC regime.^57^ Consequently, the space charge limited current under these circumstances can be stated as follows:

$$J_{\mathrm{SCLC}}=\frac{9\mu_{\mathrm{eff}}\varepsilon_{0}\varepsilon_{\mathrm{Terp}-Fc}}{8}\frac{V^{2}}{d^{3}}$$ 13

The electron’s mobility in the organic layer interfacial is represented by μ_eff_. ε_Terp‑Fc_ = 3.05 is the permittivity of the Terp-Fc organic material computed by the DFT method, and the thickness (*d*) of the interfacial Terp-Fc organic layer is calculated by using SEM to be 55 nm. The current density (*J*)-voltage (*V*)^2^ graphs of the Al/Terp-Fc/p-Si diode are shown in Figure 14a at different temperatures. The μ_eff_ values were calculated from the temperature-dependent graphs. For example, it was computed that the value of μ_eff_, as determined by eq 13, was 7.72 × 10^–14^ m^2^ V^–1^ s^–1^ for 300 K. Table 3 provides a summary of the calculated charge carrier mobility values of the produced diodes at the measured temperatures. The findings demonstrate that raising the temperature causes the carriers’ mobility in the Al/Terp-Fc/p-Si diode to increase. This increase is indicative of thermally assisted hopping transport, commonly observed in organic semiconductors. Improved mobility at higher temperatures reduces the transit time and increases the diffusion length of carriers, ultimately enhancing the diode’s overall conductivity and efficiency.

**Figure 14:**
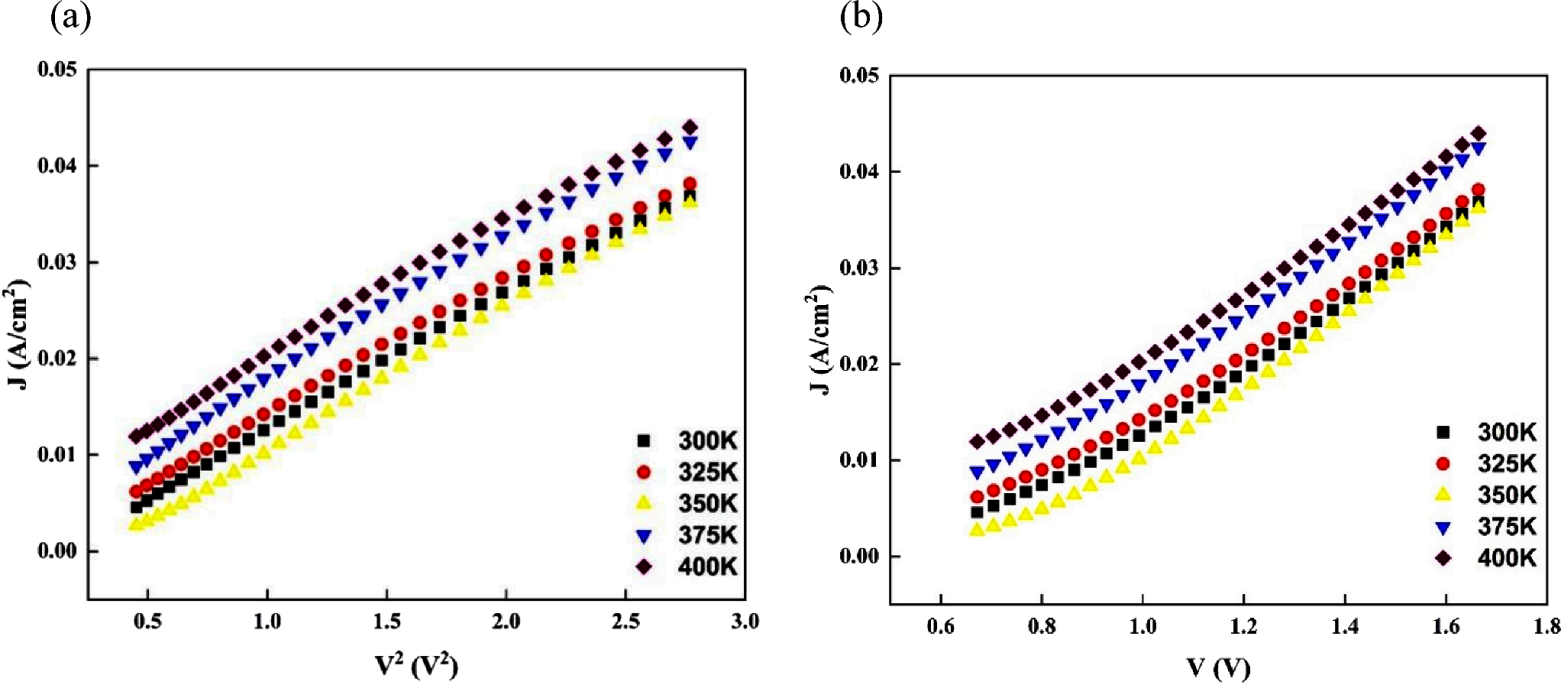
(a) *J* versus *V* ^2^ graphs of Al/Terp-Fc/p-Si diode in the temperature range from 300 to 400 K. (b) *J* versus *V* graphs of Al/Terp-Fc/p-Si diode in the temperature range from 300 to 400 K.

Figure 14b shows the graphs of *J* vs *V* of the Terp-Fc/p-Si diode at different temperatures. The slope values at each temperature were used to determine the transit time (τ) values. These τ values were used to calculate the diffusion length (*L*_*D*_) values. Also, μ_eff_ was used to calculate the diffusion coefficient (*D*). Table 4 provides a summary of the calculated *D*, *L*_*D*_, and τ values of the produced diodes at the measured temperatures. The slope of region I of the linear region of ln*I* vs ln*V* indicates the temperature-induced effective conductivity (σ), which is clearly visible. It is evident from Table 4 that as the measured temperature rises, so does the effective conductivity and charge carrier concentration close to the junction of the device.

## 4 Conclusions

A study was reported on the temperature-dependent diode, impedance, and dielectric characteristics of applying a Terp-Fc organic layer via spin coating on p-Si surfaces. Some of the dielectric parameters studied are the real and imaginary electrical moduli (*M*′, *M*″), dielectric constant (ε′), loss tangent (tanδ), and ac electrical conductivity (σ_AC_) of the Terp-Fc/p-Si diode. Experimental capacitance frequency (*C* – *f*) and conductance frequency (*G* – *f*) measurements were used to examine these parameters over a temperature range of 300–400 K and a frequency range of 0.3–1.5 MHz. In the same frequency and temperature ranges, impedance properties were also examined. According to experimental results, the impedance and dielectric characteristics exhibit strong temperature and frequency dependence. The results of σ_AC_ suggest that the conduction process aligns with the CBH theory in the temperature range from 300 to 350 K and NPST in the temperature range from 350 to 400 K. The diode’s impedance spectroscopy characteristics were examined at frequencies between 300 Hz and 1.5 MHz and in the temperature range from 300 to 400 K. Semicircles appeared on Nyquist’s plots of the diode, showing high electrical device performance and thermal stability over the measured frequency and temperature ranges. We proposed an electrical equivalent circuit for the produced diode, and the main fitting parameters were determined according to this circuit.

The temperature-dependent *I*-*V* measurements demonstrated excellent diode performance with an ideality factor of 2.87–2.02, a barrier height of 0.704–0.871 eV, a high shunt resistance of 746–79 kΩ, a low series resistance of 353–383 Ω, and a high RR of 600–70, at various temperatures ranging from 300 to 400 K. According to the temperature-dependent current–voltage characteristics of the fabricated diode, three main current conduction mechanisms are defined in the positive bias region: thermionic emission region (for *V* < 0.128 V), the TCLC region due to an exponential distribution of traps (for 0.16 ≤ *V* < 0.64 V), and SCLC region controlled by a single trap state (for 0.672 ≤ V ≤ 2 V). It is also observed that the reverse leakage current is dominated at all temperatures by the Schottky emission at the measured voltage region. Furthermore, the presence of dual activation energies and the temperature-dependent enhancement in carrier mobility strongly suggest that the Terp-Fc/p-Si interface exhibits stable, tunable electronic behavior. These features make the proposed diode architecture highly promising for flexible, thermally sensitive, or sensor-oriented applications in organic semiconductor technologies.

The Terp-Fc displayed a semiconducting energy bandgap determined with the DFT method, enabling an organic–inorganic semiconductor device (Terp-Fc/p-Si). The Al/Terp-Fc/p-Si/Al diode configuration highlights the importance of the Terp-Fc interfacial layer in semiconductor technology and shows possibilities for future applications in electrical devices.

## Supplementary Material



## Notes

DFT-based computational analyses and Hall measurements of the Terp-Fc compound; molecular orbital properties, energy gap, and reactivity descriptors calculated to confirm the semiconductor nature of Terp-Fc; Hall effect measurements further substantiate its *n*-type conductivity, providing carrier mobility and sheet carrier density values (PDF)
