# Supplementary material for: Exploring Charge Transport Mechanisms and Dielectric Spectroscopy in Terp-Fc Layers Schottky Diodes under Temperature Variations
Source: ACS Omega. 2025 May 8;10(19):19510–21. doi: 10.1021/acsomega.5c00033 (PMC12096249; doi:10.1021/acsomega.5c00033)
Supplement: Supplementary file 1 [file ao5c00033_si_001.pdf]

Supplementary information

Exploring Charge Transport Mechanisms and Dielectric Spectroscopy in Terp-Fc Layers Schottky Diodes under Temperature Variations

Pınar Oruç [a*], Ali Osman Tezcan [b], Serkan Eymur [c], Nihat Tuğluoğlu [c]

[a] Department of Physics, Faculty of Sciences, Gazi University, Ankara, Turkey

[b] Department of Electricity and Energy, Şebinkarahisar Vocational School, Giresun University, Giresun, Turkey

[c] Department of Energy Systems Engineering, Faculty of Engineering, Giresun University, Giresun, Turkey

* Corresponding author.

E-mail address: pinaroruc@gazi.edu.tr (P. Oruç)

S1. Computational studies

The synthesized Terp-Fc compound was first subjected to a DFT-based computational study, and the molecular geometry of the compound was theoretically optimized. In this way, detailed information about the Terp Fc compound's electronic structure, conductivity, and ground state was obtained. The optimized molecular structure was utilized to conduct simulations of the highest occupied molecular orbital (HOMO) and lowest unoccupied molecular orbital (LUMO) using GaussView5 software [28]. Figure S1 displays the visual depiction of HOMO, LUMO orbitals, and orbital energy levels. Figure S1 demonstrates that the energy difference between the HOMO and the LUMO levels is 3.48 electron volts (eV). According to the literature, since semiconductors' energy band gap (ΔE_g) is typically in the range of 0.5-4.0 eV, the determined ΔE_g value of Terp-Fc may indicate that it may exhibit semiconductor properties. The descriptors of molecular global reactivity (electron affinity, ionization potential, chemical

hardness, chemical softness, electronegativity, chemical potential, etc.), which are determined by the calculated HOMO and LUMO values, are listed in Table S1.

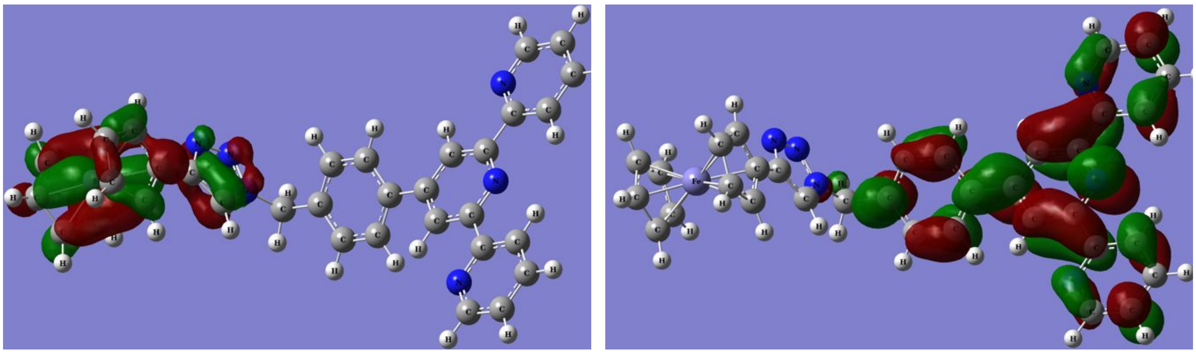

E_{HOMO} = -5.084 eV E_{LUMO} = -1.598 eV

$\Delta E = |E_{HOMO}-E_{LUMO}|$ = 3.486 eV

Figure S1. DFT computed energy levels of the HOMO and LUMO of Terp-Fc.

Table S1. Some computed molecular descriptors depend on the HOMO and LUMO energy values of the compound.

Parameters (eV)	Value
E_{LUMO}	-1.598
E_{HOMO}	-5.084
Energy band gap $\|E_{HOMO}–E_{LUMO}\|$	3.486
Ionization potential ($I = –E_{HOMO}$)	5.084
Electron affinity ($A = –E_{LUMO}$)	1.598
Chemical hardness ($\eta = (I–A)/2$)	1.743
Chemical softness ($\zeta = 1/2\eta$)	0.287
Electronegativity ($\chi = (I+A)/2$)	3.341
Chemical potential ($\mu = –(I+A)/2$)	-3.341
Electrophilicity index ($\omega = \mu^2/2\eta$)	3.202
Maximum charge transfer index ($\Delta N_{max.} = –\mu/\eta$)	1.917

S2. Hall Measurement

The Hall measurement analysis of the Terp-Fc sample confirms its classification as an n-type semiconductor, as indicated by the negative Hall voltage, which signifies electron conduction as the dominant charge transport mechanism. The measured Hall mobility is 166.65 $cm^2/V{\cdot}s$,

suggesting a moderate level of carrier transport efficiency. Furthermore, the sheet carrier density is 9.86×10^{10} cm^{-2}, indicating a moderately doped material with a free carrier concentration suitable for electronic applications. The observed mobility and carrier concentration values may be influenced by factors such as crystallographic properties, doping levels, and potential interfacial effects. These findings highlight the potential of Terp-Fc for integration into semiconductor-based electronic and optoelectronic devices.
